# Supplementary material for: Single-Cell Analysis of ADSC Interactions with Fibroblasts and Endothelial Cells in Scleroderma Skin
Source: Cells. 2023 Jul 5;12(13):1784. doi: 10.3390/cells12131784 (PMC10341100; doi:10.3390/cells12131784)
Supplement: Supplementary file 1 [file cells-12-01784-s001.zip › cells-2457600-supplementary.pdf]

Supplementary Data

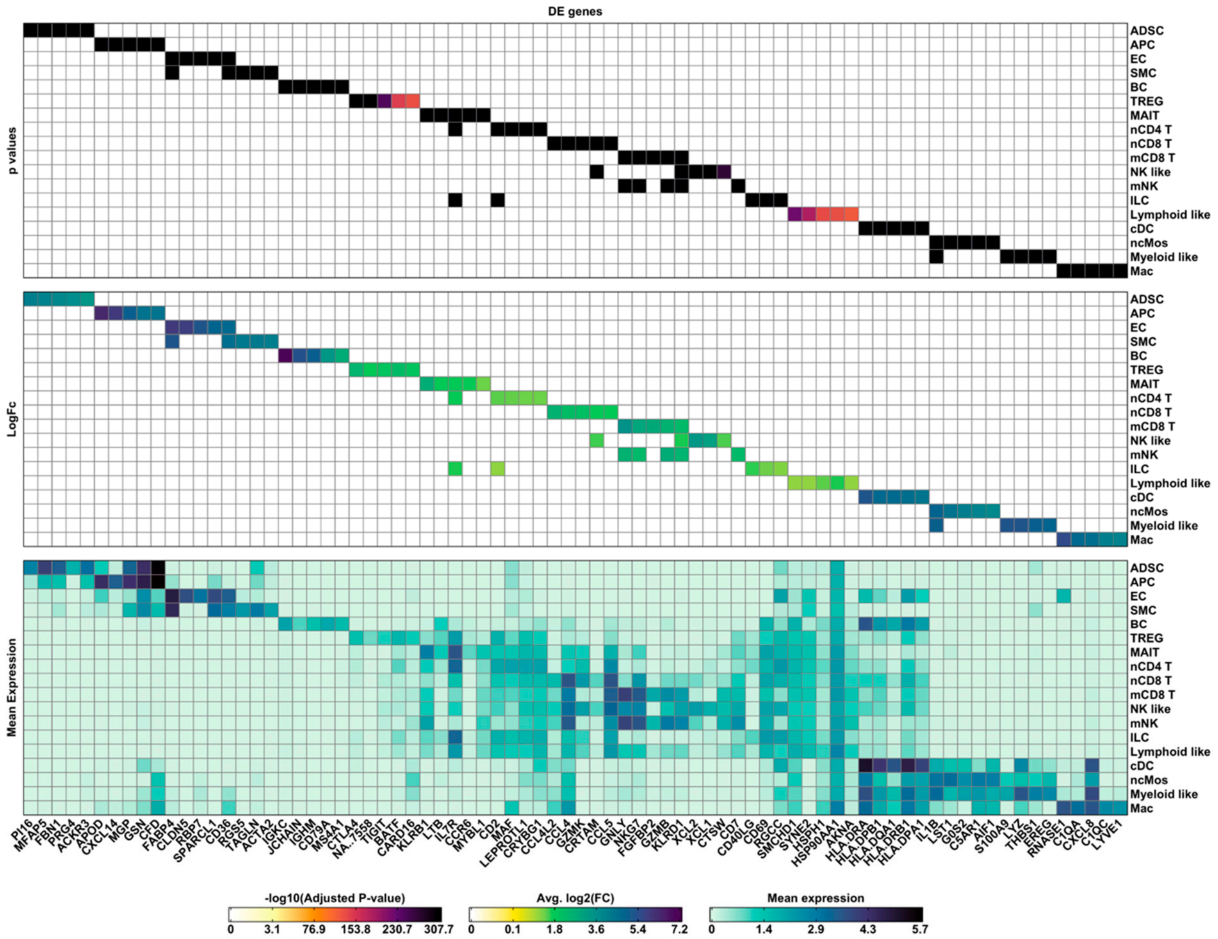

Figure S1. Differentially expressed genes (DEGs) support the clustering of the stromal vascular fraction (SVF). A three-section heatmap showing the top 5 marker genes for each cluster and their mean expression (*bottom*), the associated logFC value (*middle)* and the associated p-value (*top*). These DEGs support the clustering seen in Figure 1.

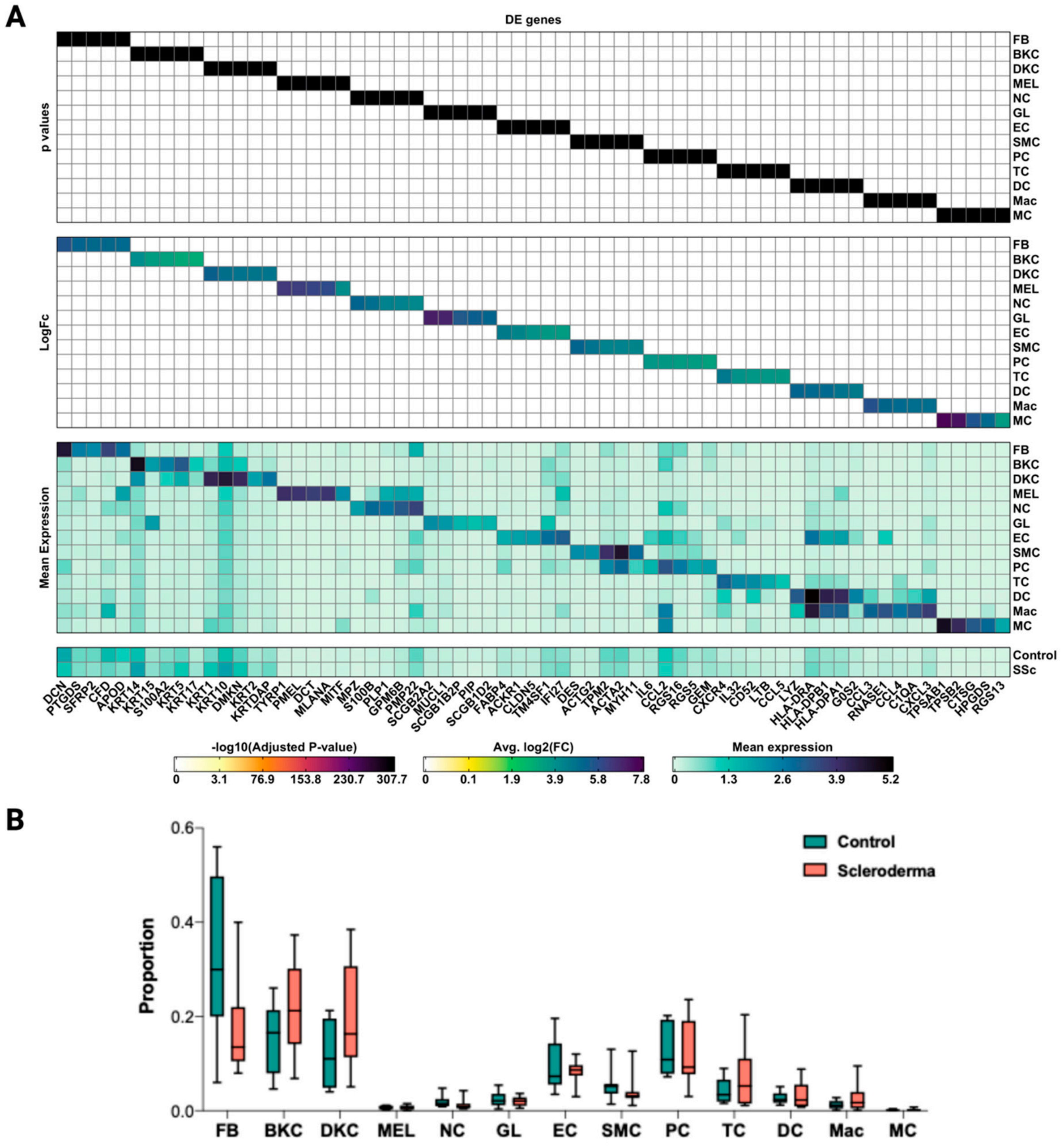

Figure S2. Differentially expressed genes (DEGs) support the clustering of control and scleroderma skin biopsies. (A) A three-section heatmap showing the top 5 marker genes for each cluster with their mean expression per cluster and compared between control and scleroderma (*bottom*), the associated logFC value (*middle)* and the associated p-value (*top*). These DEGs support the clustering seen in Figure 3. **(B)** Boxplots showing the proportion of cells derived from the skin of scleroderma (n = 12) and control (n = 10) for each cell type. * indicates significance ($p < 0.05$). Multiple unpaired t-tests were used for analysis.

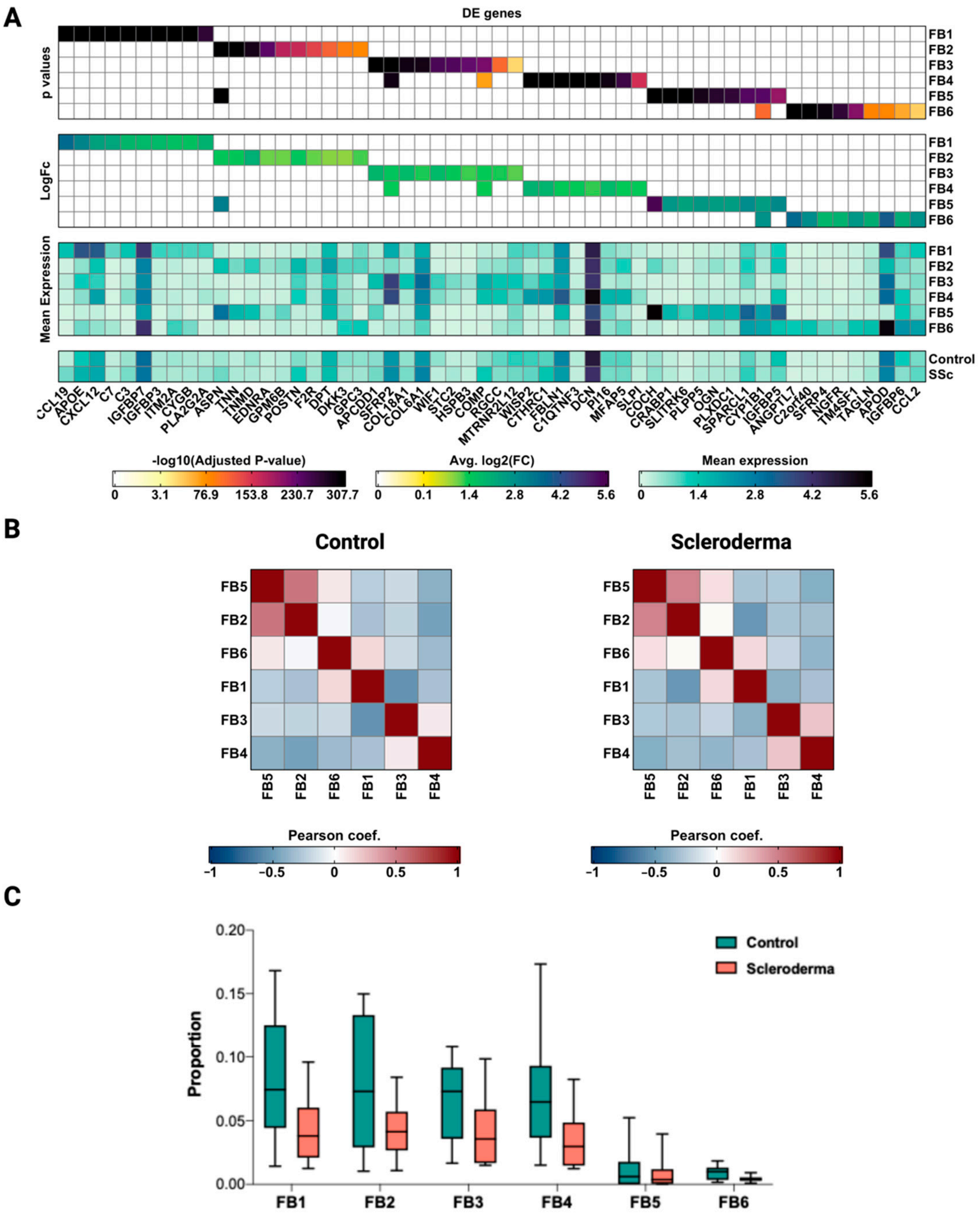

Figure S3. Marker genes of fibroblast subsets and further evidence for sub-clustering approach. (A) A three-section heatmap showing the top 5 marker genes for each cluster with their mean expression per cluster and compared between control and scleroderma (*bottom*), the associated logFC value (*middle)* and the associated p-value (*top*). These DEGs support the

clustering seen in Figure 4. **(B)** Correlation matrices based on Pearson's product-moment correlation coefficient (r) exploring the overlap of highly variable genes between the clusters. Red colour indicates maximum overlap. Blue colour indicates minimum overlap. **(C)** Boxplots showing the proportion of cells derived from the skin of scleroderma (n = 12) and control (n = 10) for each cell type. * indicates signficance ($p < 0.05$). Multiple unpaired t-tests were used for analysis.

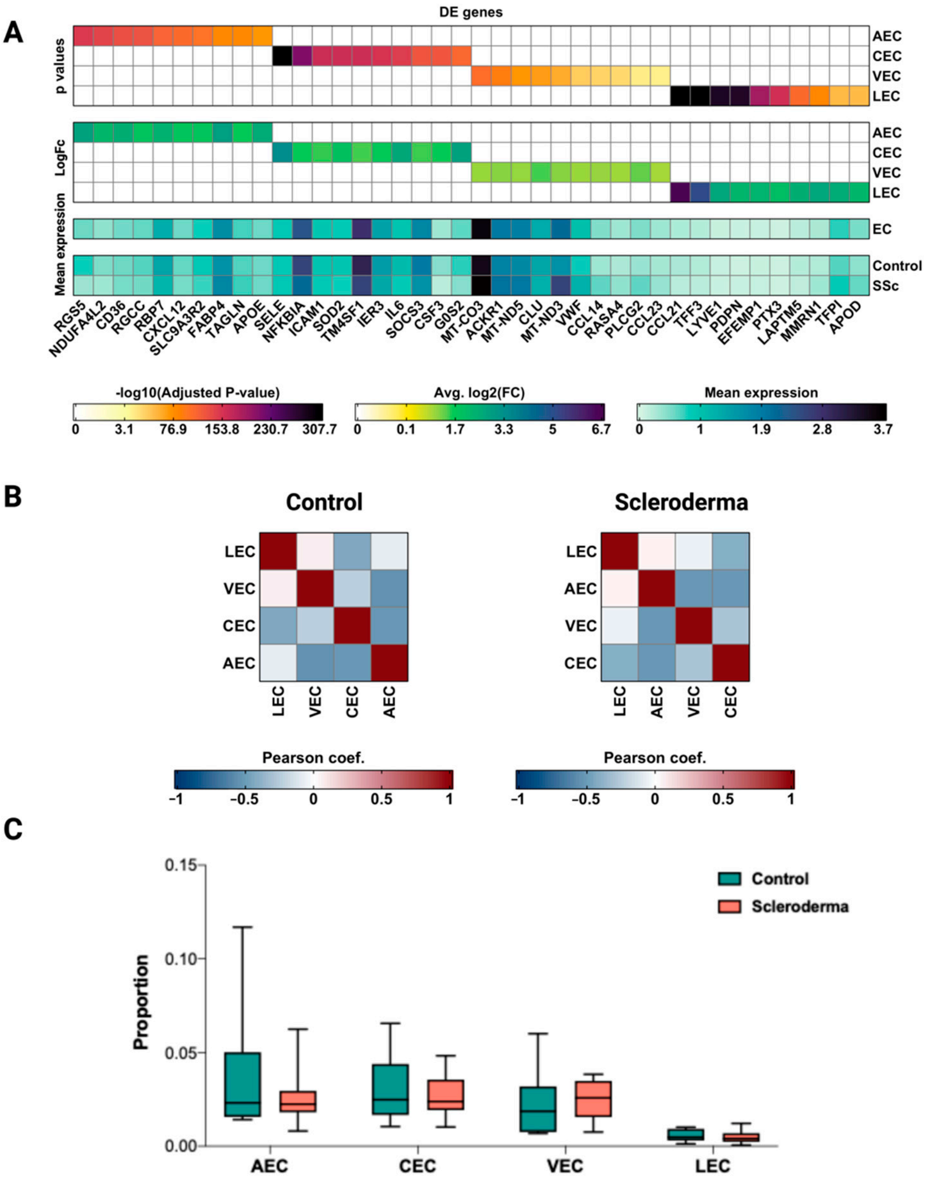

Figure S4. Differentially expressed genes of endothelial subsets and further evidence for sub-clustering approach. (A) A three-section heatmap showing the top 5 marker genes for each cluster with their mean expression per cluster and compared between control and scleroderma (*bottom*), the associated logFC value (*middle)* and the associated p-value (*top*). These DEGs support the clustering seen in Figure 5. **(B)** Correlation matrices based on Pearson's product-moment correlation coefficient (r) exploring the overlap of highly variable genes between the clusters. Red colour indicates maximum overlap. Blue colour indicates minimum overlap. **(C)** Boxplots showing the proportion of cells derived from the skin of scleroderma (n = 12) and control (n = 10) for each cell type. * indicates signficance ($p < 0.05$). Multiple unpaired t-tests were used for analysis.

<table>
<tr><th></th><th colspan="2">No change</th><th colspan="2">Additional (+)</th><th colspan="2">Missing (-)</th></tr>
<tr><td>FB1</td><td>CCN2|EGFR
CCN1|ITGB5
CCN2|LRP1</td><td>FNDC5|ITGB5
PTHLH|RAMP2</td><td>TNFSF9|HLA-DPA1
CCN1|CAV1</td><td>CCN1|ITGAV
FNDC5|ITGAV</td><td>SERPINE1|ITGB5</td><td></td></tr>
<tr><td>FB2</td><td>DKK1|KREMEN1
CCN1|ITGB5
CCN2|LRP1</td><td>FNDC5|ITGB5
PTHLH|RAMP2</td><td>CD55|ADGRE2
VEGFD|NRP2
RARRES1|NRP2
CCN1|CAV1</td><td>TNXB|SDC1
FGF2|SDC1
CCN1|ITGAV
FNDC5|ITGAV</td><td></td><td></td></tr>
<tr><td>FB3</td><td>CCN1|ITGB5
CCN2|LRP1</td><td>FNDC5|ITGB5</td><td>CCN1|CAV1</td><td></td><td>SERPINE1|ITGB5
DKK1|KREMEN1</td><td></td></tr>
<tr><td>FB4</td><td>DKK1|KREMEN1
CCN2|EGFR
CCN1|ITGB5</td><td>CCN2|LRP1
FNDC5|ITGB5
CXCL12|ACKR3</td><td>CCN1|CAV1
CCN1|ITGAV</td><td>FNDC5|ITGAV
ADM|ACKR3</td><td>CSF1|SIRPA
SERPINE1|ITGB5</td><td>PTHLH|RAMP2</td></tr>
<tr><td>FB5</td><td>RARRES1|NRP
VEGFD|NRP2
DKK1|KREMEN1
PTHLH|PTH1R</td><td>TNXB|SDC1
FGF2|SDC1
PTHLH|RAMP2</td><td></td><td></td><td>VEGFA|NRP2
BMP7|PTPRK
COL6A6|SDC1</td><td>HSPG2|SDC1
MYOC|FZD1
SERPINE1|ITGB5</td></tr>
<tr><td>FB6</td><td>RARRES|NRP2
VEGFD|NRP2
LAMC1|ITGA6
ADAM9|ITGA6
CCN2|EGFR</td><td>LAMB2|ITGA6
FN1|ITGA6
CCN2|LRP1
PTHLH|RAMP2</td><td>WNT11|FZD2
THBS1|ITGA6</td><td>THBS2|ITGA6</td><td>VEGFA|NRP2
JAG1|NOTCH3</td><td>CCN2|LRP6
MYOC|FZD1</td></tr>
</table>

Figure S5. Differences in interactions between control and scleroderma fibroblasts. Table showing the analysis of comparing Figure 6A,B, which display the top 25 interactions between ADSCs (ligands) and fibroblast subsets (receptors). Interactions are shown as "Ligand | Receptor". "No change" indicates that the pair appears in the equivalent control as well as the

scleroderma subset. "Additional (+)" indicates that the pair only appears in the scleroderma subset. "Missing (-)" indicates that the pair only appears in the control subset.

	No change		Additional (+)		Missing (-)	
**AEC**	THBS2|CD36<br>COL1A1|CD36<br>PRG4|CD44<br>COL6A6|CD44<br>VCAN|CD44<br>COL14A1|CD44	HAS2|CD44<br>C3|CD81<br>ANOS1|FGFR1<br>GPC3|CD81<br>MMP2|FGFR1	COL1A2|CD36<br>TNFSF4|TNFRSF4	PTHLH|ADRB2<br>LAMA2|CD44	THBS2|NOTCH3<br>COL6A3|CD44<br>COL1A1|CD44	COL6A2|CD44<br>VEGFA|CD44
**CEC**	VEGFD|NRP2<br>THBS2|CD36<br>COL1A1|CD36<br>VCAN|SELP	C3|CD81<br>GPC3|CD81<br>ANOS1|FGFR1<br>MMP2|FGFR1	COL1A2|CD36<br>PTHLH|ADRB2<br>PRG4|CD44<br>COL14A1|CD44<br>COL6A6|CD44	LAMA2|CD44<br>VCAN|CD44<br>HAS2|CD44<br>ANOS1|FGFR1	RARRES1|NRP2	VEGFA|NRP2
**VEC**	VEGFD|NRP2<br>VCAN|SELP	C3|CD81<br>GPC3|CD81	PRG4|CD44<br>COL14A1|CD44<br>COL6A6|CD44	LAMA2|CD44<br>VCAN|CD44<br>HAS2|CD44	RARRES1|NRP2	VEGFA|NRP2
**LEC**	VEGFD|NRP2<br>C3|CD81<br>ANOS1|FGFR1	GPC3|CD81<br>MMP2|FGFR1	NTN1|UNC5B<br>COL1A1|FLT4<br>COL1A2|FLT4<br>TNFSF4|TNFRSF4<br>PRG4|CD44	COL14A1|CD44<br>COL6A6|CD44<br>LAMA2|CD44<br>HAS2|CD44	RARRES1|NRP2<br>VEGFA|NRP2<br>THBS2|CD36	COL1A1|CD36<br>VCAN|SELP

Figure S6. Differences in interactions between control and scleroderma endothelial cells. Table showing the analysis of comparing Figure 6A,B, which display the top 25 interactions between ADSCs (ligands) and endothelial cell subsets (receptors). Interactions are shown as "Ligand | Receptor". "No change" indicates that the pair appears in the equivalent control as well as the scleroderma subset. "Additional (+)" indicates that the pair only appears in the scleroderma subset. "Missing (-)" indicates that the pair only appears in the control subset.

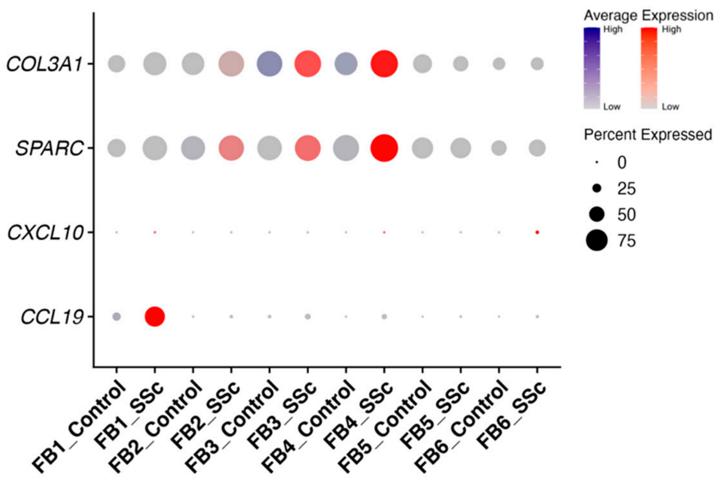

Figure S7. Expression of proinflammatory markers in fibroblasts. Dot plot of genes associated with a proinflammatory phenotype by Korsunsky*, et al.* [9]. Colour intensity implies level of expression and dot size indicates the percentage of the cluster expressing the gene (blue = control; red = scleroderma).
